# Ovarian and Endometrial Endometrioid Carcinoma Following the Use of a Biologic IL-17 Inhibitor

**DOI:** 10.7759/cureus.42481

**Published:** 2023-07-26

**Authors:** Luke Babcock, Samantha R Singer, Pamela Carbiener

**Affiliations:** 1 Family Medicine, Halifax Health Medical Center, Daytona Beach, USA; 2 Obstetrics and Gynecology, Florida State University College of Medicine, Tallahassee, USA; 3 Obstetrics and Gynecology, Halifax Health Medical Center, Daytona Beach, USA

**Keywords:** accelerated carcinogenesis, secukinumab, perimenopausal, endometrial carcinoma, ovarian carcinoma, biologic il-17 inhibitor

## Abstract

Evidence suggests that IL-17, a pro-inflammatory cytokine, suppresses tumor carcinogenesis; therefore, the use of IL-17 inhibitors accelerates carcinoma growth. We present a case of a perimenopausal female who was diagnosed with synchronous primary ovarian and endometrial endometrioid carcinoma following the use of secukinumab, a monoclonal antibody against IL-17. After eight months of secukinumab, she developed progressive vaginal bleeding, left upper quadrant pain, and abdominal distention. CT imaging displayed a large abdominal mass, and biopsies produced the diagnosis. It is proposed that by inhibiting IL-17, carcinogenesis was expedited. This case highlights a relationship between secukinumab and accelerated carcinogenesis. Consequently, due to the incidence of endometrial carcinoma and the morbidity rate of ovarian carcinoma, individuals taking IL-17 inhibitors may need prophylactic screening and close monitoring.

## Introduction

Ovarian cancer is the most lethal gynecologic cancer, with more than 50% of females passing within five years of diagnosis [1]. The median age at diagnosis is 63 years old, with 17.5% of all new cases diagnosed between the ages of 45 and 54 [1]. The major risk factors for epithelial ovarian carcinoma include age over 50, with every year past 50 conferring an 11% increase in relative risk [2], menarche before age 12, menopause after age 52, nulliparity [3], family history, genetic factors including breast cancer gene 1 (*BRCA1*) and *BRCA2* [4], endometriosis [5], and asbestos exposure [6].

Secukinumab is a human-derived monoclonal antibody that binds IL-17A, a pro-inflammatory cytokine implicated in autoimmune conditions such as psoriasis, psoriatic arthritis, and ankylosing spondylitis. The most common adverse reactions to secukinumab, per phase lI and III clinical trials [7,8], are upper respiratory tract infections, nasopharyngitis, headaches, and diarrhea. There is some evidence that biologic agents increase the risk of malignancy [9-12]. For example, the risk of non-melanoma skin cancer is increased by taking biologic inhibitors of tumor necrosis factor (TNF)-alpha [9]. Moreover, until further research comprehensively examines an exhaustive list of consequences, prescribers should diligently monitor and screen patients on biologic agents.

## Case presentation

A 49-year-old G0P0 perimenopausal female was referred to a gynecologic oncologist after a four-month period of progressive abdominal distention following the use of a biologic IL-17 inhibitor, secukinumab. Before starting secukinumab, she suffered from seven years of worsening joint pains, extensor scaly plaques, and eventually a confluent blistering erythemal rash covering about 90% of her body. Her condition did not respond to prednisone or methotrexate. She was enrolled in a clinical trial for the treatment of psoriasis, consisting of weekly subcutaneous abdominal injections of secukinumab. After about a month of this trial, her skin and joint findings almost completely resolved. She also at this time developed light vaginal spotting. Two months later, she developed mild periumbilical pain described as “muscle soreness.” At four months, she noticed mild left upper abdominal distension, dyspepsia, and increasing duration and amount of vaginal bleeding. Her skin and joint psoriatic symptoms also started to recur around this time. Aside from abdominal distention, she was remarkably asymptomatic and progressed for another four months until she was discontinued from secukinumab and referred to gynecology.

Her past medical history is significant for adult-onset severe psoriasis, obesity, and perimenopausal bleeding after six months of amenorrhea prior to secukinumab. She denied a family history of ovarian, endometrial, breast, or any type of cancer. She had a 35-pack-year smoking history but quit two years ago.

On physical examination, her vital signs were within normal limits. She had a markedly distended, firm, large, smooth-walled abdominal mass extending superiorly to the sternum and laterally from side wall to side wall. The mass demonstrated limited mobility. She had no rebound tenderness or guarding. During abdominal auscultation, distant bowel sounds were detected, providing additional support to the suspicion of a present mass.

**Figure 1:**
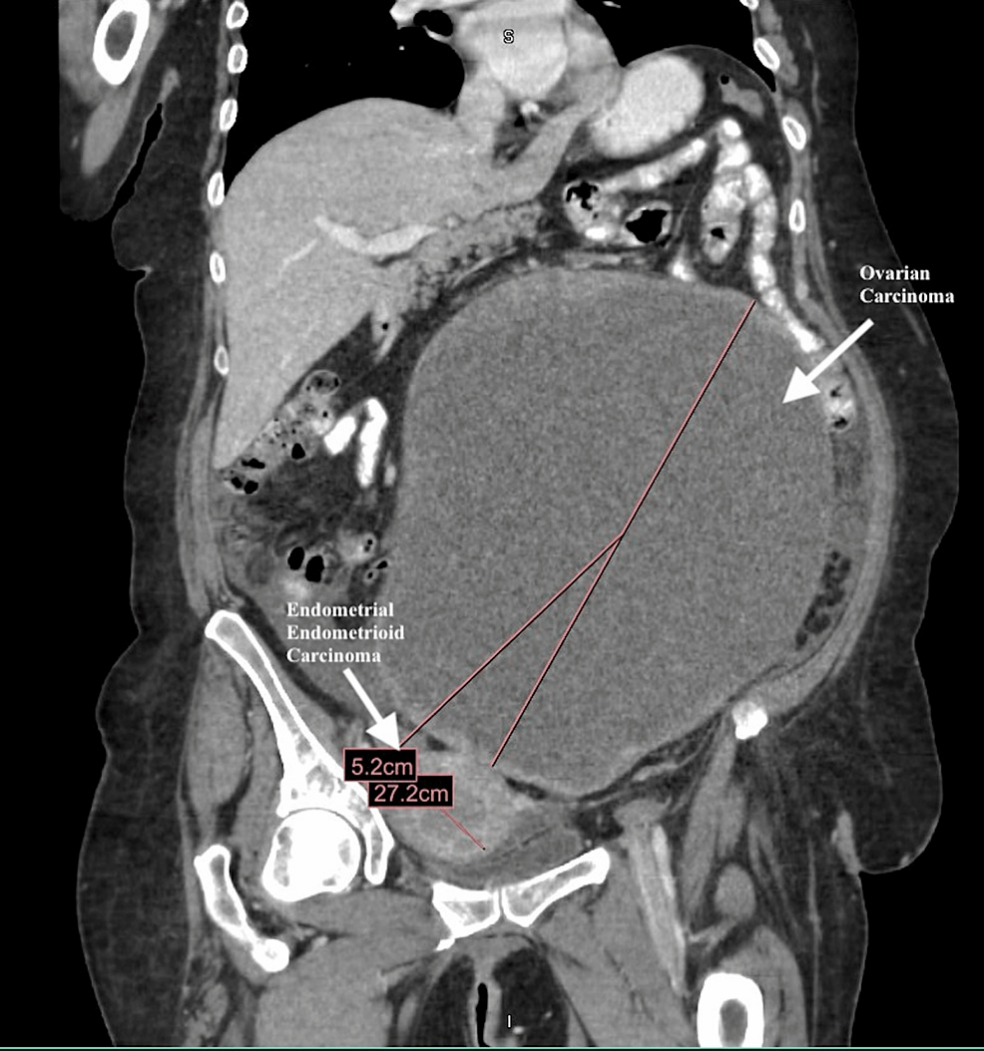
Abdominal CT scan presenting the 27 cm diameter cystic mass in greatest dimension (arrow). Her uterus is also prominent with a diameter of 5.2 cm (arrow). CT: computed tomography

**Figure 2:**
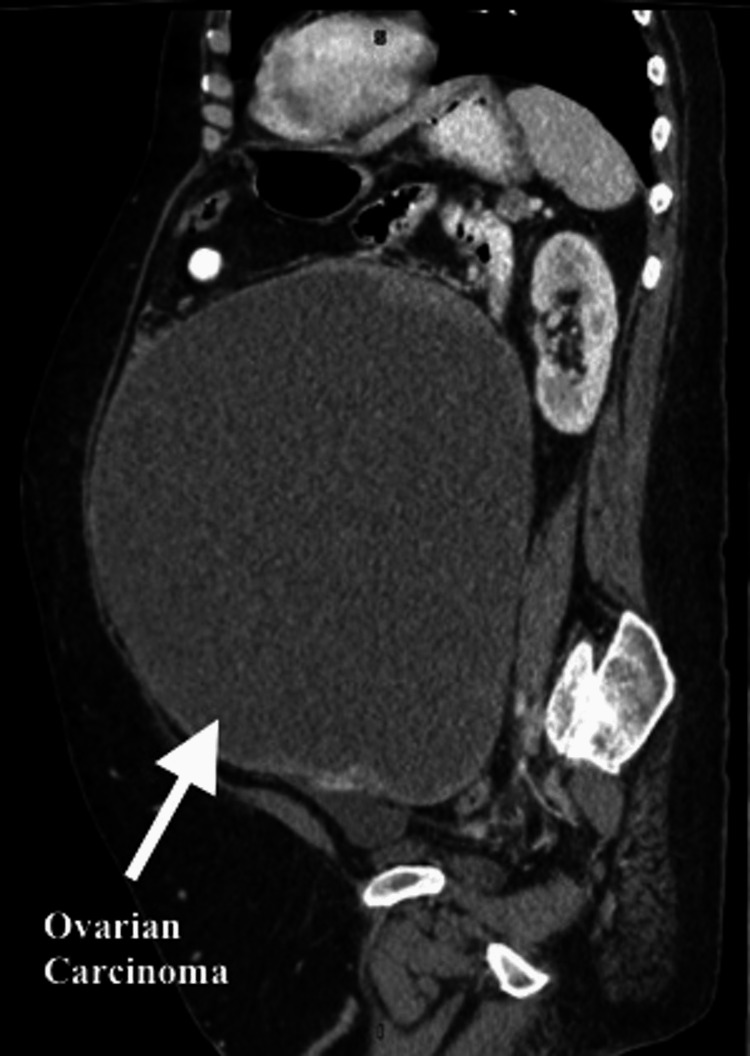
Sagittal view of the patient’s abdominal CT scan illuminating the severity of tumor size (arrow). CT: computed tomography

She had an ultrasound and abdominal CT scan, which showed in greatest dimension an extremely large, 27 cm diameter, cystic mass and some solid-appearing material around the periphery (Figures 1, 2). Her uterus was prominent, and her endometrial cavity was dilated at 12 mm with an external diameter of 5.2 cm. There was a small amount of ascites in the peritoneal cavity. Cancer antigen 125 (CA-125) was elevated at 1,381 units/mL. She underwent an endometrial biopsy, which revealed grade 1 endometrial cancer.

We explained to our patient that surgery and systemic chemotherapy are the cornerstone treatments with the possibility of an intermediate step involving fluid drainage of the mass. We also discussed significant areas of concern regarding surgery, anesthesia, autoimmune problems, severe life-threatening flare-ups, and hospitalizations, along with a host of other related issues. It was highly recommended to discontinue the use of her biologic IL-17 inhibitor and move her care to a tertiary center due to the complexity of her case. Our patient has discontinued secukinumab and continues to monitor her disease process.

## Discussion

Ovarian surface epithelium refers to a single layer of flat, cuboidal cells that overlie the ovaries [13]. It is contiguous with the pelvic mesothelium and is considered a modified version of it. Among the ovarian surfaces, epithelial tumors are the endometrioid tumors, which microscopically resemble endometrial tissue. Endometrioid tumors comprise about 15% of the total epithelial ovarian cancer incidence [2]. Normal endometrial tissue forms from the Müllerian ducts, which originate from invaginations of the embryologic ovarian surface epithelium. The ovarian surface epithelium is particularly susceptible to metaplasia, a process by which an external stressor causes de-differentiation into a separate tissue type. Ovulation requires the rupture of the ovarian surface epithelium, the release of an oocyte, and subsequent healing via the proliferation of surface epithelial cells [13]. During the healing process, ovarian surface epithelial cells act similarly to pluripotent stem cells, transforming into mesenchymal fibroblasts in addition to proliferating. Disruption of intercellular connections between ovarian surface epithelial cells during rupture releases reactive oxygen species, which can result in damage to the cells’ DNA [14]. It is theorized that this frequent exposure to oxidants increases the ovarian surface epithelium’s likelihood of metaplastic, and subsequent neoplastic, transformation into various mesothelial derivatives (e.g., serous, mucinous, transitional, squamous, endometrioid).

There is a growing body of evidence that suggests that IL-17, a pro-inflammatory cytokine, suppresses tumor carcinogenesis [15-18]. In a retrospective cohort study of 201 ovarian cancer patients, researchers found IL-17 levels in ascitic fluid to be positively correlated with survival and negatively correlated with advanced disease [16]. In another study, a higher density of IL-17-expressing cells in ovarian neoplasms correlated with lower-grade tumors, and the expression of IL-17 was independently a positive predictor for progression-free survival [17]. A retrospective cohort study of 71 ovarian cancer patients also found IL-17 levels in the peritoneal fluid to be positively correlated with survival [18].

The role of IL-17 in tumorigenesis is complex, as there is evidence for both pro-tumor and anti-tumor effects [19]. One of the functions of IL-17 is to maintain tissue integrity via the induction of tight junction proteins [20]. One theory is that by blocking IL-17, the proper expression of tight junctions during the repair phase of the ovulatory cycle is disrupted, resulting in increased exposure to reactive oxygen species.

Interestingly, our patient was found to have foam cells in her cul-de-sac and appendix that did not stain for fungi or tuberculosis. Foam cells are thought of as M2 macrophages that have engulfed lipid particles usually signifying the presence of extracellular bacterial and fungal pathogens. IL-17-producing cells in the tumor microenvironment are largely regulated M2 macrophages known as tumor-associated macrophages [20]. It is possible that the disruption of IL-17 signaling pathways led to a buildup of these regulatory macrophages, manifested as foam cells.

The revised Bethesda guidelines [16] for testing tumors for microsatellite instability (MSI) include any patient with synchronous primary hereditary nonpolyposis colorectal cancer-associated tumors, which include endometrial and ovarian cancer [17]. We, therefore, considered this to be a diagnosis and ordered for the tumor to be tested for abnormal mismatch repair proteins.

## Conclusions

We presented a case of ovarian and endometrial endometrioid carcinoma following the use of secukinumab, a monoclonal antibody against IL-17. With ovarian cancer’s high morbidity rate and the most common gynecologic cancer being endometrial carcinoma, providers for women’s health must become cognizant of the risk factors associated with the use of biologic agents such as IL-17 inhibitors. These immunomodulators have become a mechanism of treatment with such profound efficacy for diseases such as psoriasis that patients have a necessity to stay on them. Further exploration and extensive research are needed to understand the relationship between accelerated carcinogenesis and biologic agents. As more patients are prescribed immunomodulators, close monitoring and screening for risk factors may be required.
